# Organic pollution of rivers: Combined threats of urbanization, livestock farming and global climate change

**DOI:** 10.1038/srep43289

**Published:** 2017-02-23

**Authors:** Yingrong Wen, Gerrit Schoups, Nick van de Giesen

**Affiliations:** 1 Department of Water Management, Delft University of Technology, Stevinweg 1, 2628CN, Delft, The Netherlands

## Abstract

Organic pollution of rivers by wastewater discharge from human activities negatively impacts people and ecosystems. Without treatment, pollution control relies on a combination of natural degradation and dilution by natural runoff to reduce downstream effects. We quantify here for the first time the global sanitation crisis through its impact on organic river pollution from the threats of (1) increasing wastewater discharge due to urbanization and intensification of livestock farming, and (2) reductions in river dilution capacity due to climate change and water extractions. Using in-stream Biochemical Oxygen Demand (BOD) as an overall indicator of organic river pollution, we calculate historical (2000) and future (2050) BOD concentrations in global river networks. Despite significant self-cleaning capacities of rivers, the number of people affected by organic pollution (BOD >5 mg/l) is projected to increase from 1.1 billion in 2000 to 2.5 billion in 2050. With developing countries disproportionately affected, our results point to a growing need for affordable wastewater solutions.

Organic pollution of rivers by wastewater discharge from human activities (cities, farming, industry) affects humans and ecosystems worldwide through the global sanitation crisis. First, untreated urban sewage contains pathogens that cause a variety of diseases, including diarrhoea^1^, globally the leading cause of illness and death. As of 2015, up to 2.4 billion people, primarily in sub-Saharan Africa and southern Asia, lack access to proper sanitation^2^. Second, accumulation of organic pollutants in rivers stimulates microbial growth, leading to oxygen depletion and disturbance of the entire river ecosystem^3^.

The level of organic pollution in a river, commonly expressed by the Biochemical Oxygen Demand (BOD)^4^, is the result of two counteracting mechanisms: pollutant loading and natural cleaning (Fig. 1). Wastewater discharge from cities and intensive livestock farms constitute the main organic pollutant loads into rivers^5^,^6^. With rapid urban population growth expected in the next decades, both sources of organic pollution will increase^7^. Although pollution is introduced at wastewater discharge points along the river, impacts extend to downstream populations and ecosystems, as pollutants are transported through the river network^8^. The extent of downstream impacts depends on self-cleaning capacities of rivers via dilution by natural runoff and natural degradation by micro-organisms, as illustrated for two major basins in Fig. 1. Changes in river discharge due to climate change affect river dilution capacities, increasing the risk of river pollution in areas that experience reductions in climate wetness^9^,^10^. Increases in water extractions to support a growing global population may further decrease river dilution capacities.

Quantitative assessments of human and climate effects on increasing in-stream BOD have been carried out at catchment and continental scales^11^. However, climate-related organic river pollution requires a global perspective to articulate the geographic linkage of urbanization, intensive livestock farming, and freshwater variability. Global-scale studies of river BOD so far have ignored wastewater from livestock farming and self-cleaning capacities of rivers by natural degradation^12^,^13^, as well as future climate-related changes in river dilution capacity^4^. Here, we calculate for the first time historical and future in-stream BOD concentrations in global river networks, accounting for BOD loading from urban areas and intensive livestock farming, wastewater treatment, downstream transport, dilution and natural degradation. Our model is described in Section S1, with detailed information on all spatially distributed model inputs in Section S2. Comparison of computed BOD concentrations to observations from global, continental, and national river BOD datasets in Section S3 provides confidence in the presented results. In what follows, we analyse global patterns of historical (year 2000) and future (year 2050) BOD concentrations and discuss wastewater management options to curb projected increases in organic river pollution.

## Results

### Historical patterns of organic river pollution

**Figure 2:**
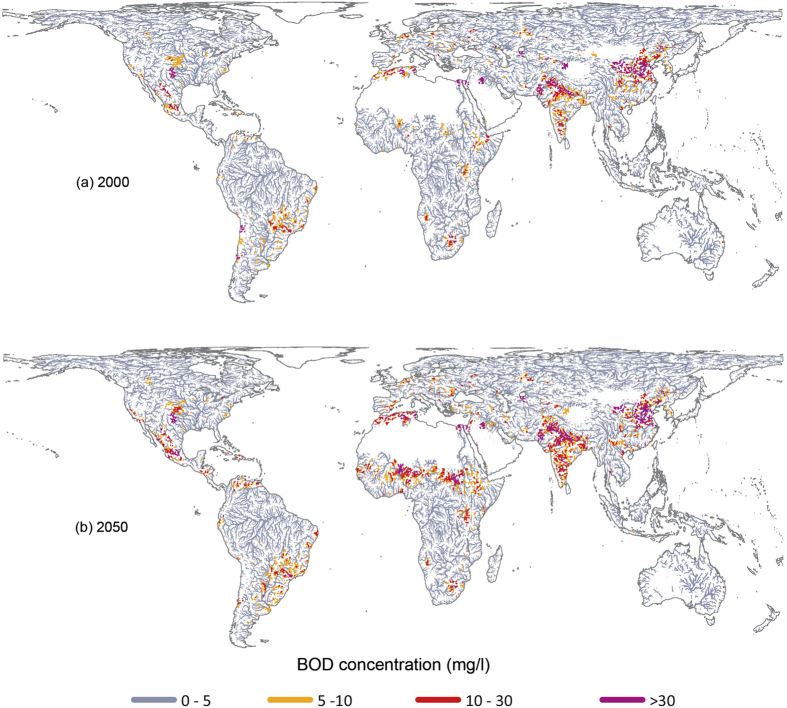
Global patterns of computed river BOD concentrations in the years 2000 and 2050^53^,^54^.

Figure 2a shows global patterns of computed BOD concentration for the year 2000 resulting from corresponding spatial patterns in river discharge (Fig. S2), urban population (Fig. S3), intensive livestock farming (Figs S4 and S5), wastewater treatment (Figs S6 and S7), and natural degradation. The impacts of intensive livestock farming are significantly more widespread than those of urban populations (about 5 times more polluted grid cells; Fig. S13). Sizeable portions (about 23% of polluted grid cells) of these organic pollutants are naturally degraded. Further reductions in BOD concentrations by wastewater treatment are successful in removing river pollution in large parts of Europe (69%) and North America (68%), while in other regions (Indian sub-continent, mid-eastern China, South Korea, Brazil, Mexico, as well as smaller regions in Africa, south-eastern Asia) wastewater treatment remains insufficient to keep BOD concentrations below 5 mg/l (Fig. 2a).

**Figure 3:**
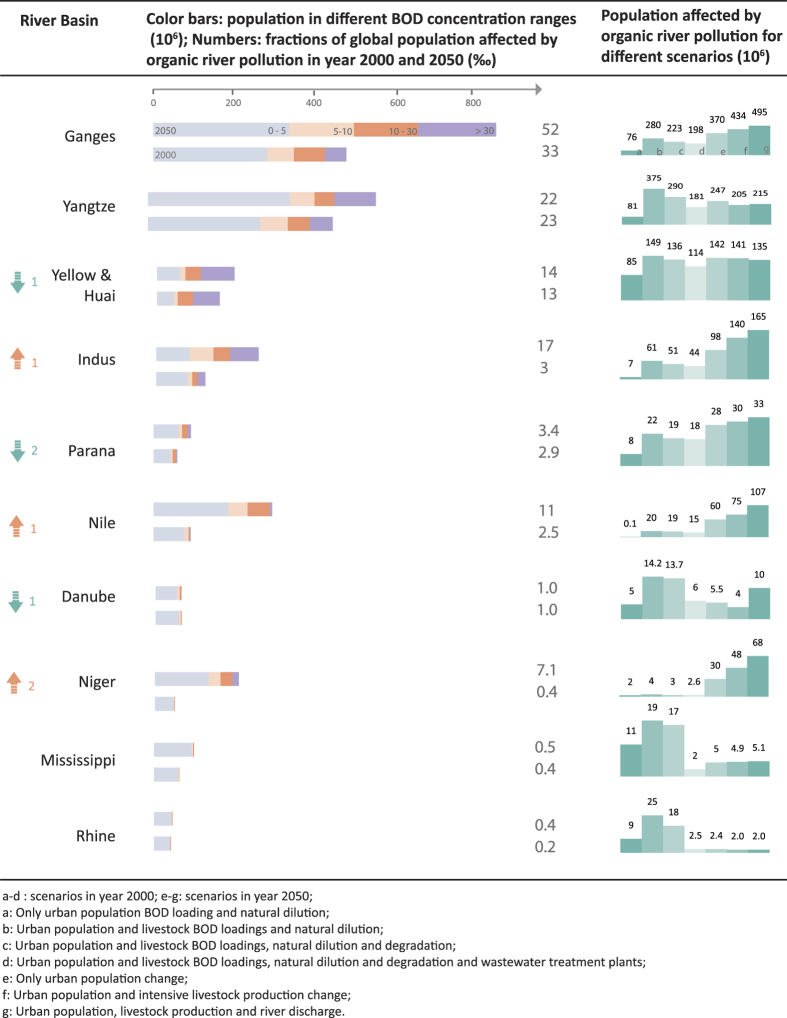
Number of people (in millions) living along polluted rivers in 10 major river basins. Basins are listed from more to less polluted in the year 2000, with green and red arrows to the left indicating their change in ranking in the year 2050.

Figure 3 summarizes the number of people living near polluted rivers for major river basins around the world, with Asian basins (Ganges, Yangtze, Yellow & Huai, Indus) topping the list. Note that about half of the affected people live in smaller basins scattered throughout the world. The numbers in Fig. 3 also reveal the main pollution sources in each basin, i.e. urban (Yellow & Huai), livestock (Ganges), or a combination of both (Yangtze). Natural degradation and wastewater treatment are most successful in reducing pollution impacts in the Rhine, Mississippi, and Danube basins.

**Figure 4:**
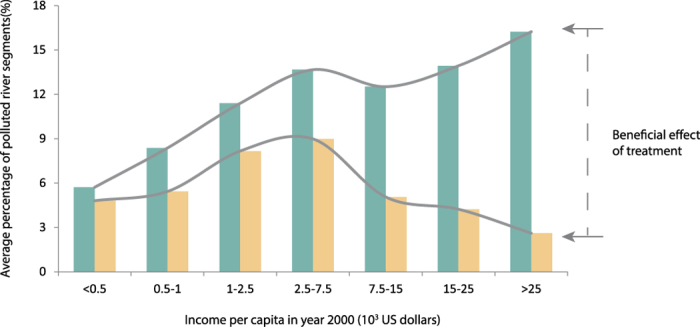
Environmental Kuznets curves for the relation between computed country-wide organic river pollution (with and without wastewater treatment) and per capita income in the year 2000.

These results largely agree with the notion of an environmental Kuznets curve^14^, as shown in Fig. 4. Relatively low levels of pollution occur in both poor and rich nations due to, respectively, absence and control of pollution sources. Relatively high levels of pollution are found in rapidly developing nations characterized by urbanizing populations and expanding economies that have not yet implemented comprehensive control and treatment of pollution sources^15^,^16^. There is however also large heterogeneity within countries. For example, China is one of the fastest growing economies in the world but its urban population and economic development are concentrated in the eastern part of the country. Likewise, organic river pollution is also concentrated in the east (Fig. 2a), despite a higher rate of 55% wastewater treatment in eastern China compared to 20% in western China^17^. While urban areas possess financial and technical resources for pollution control, control measures typically lag behind population increase.

### Future patterns of organic river pollution

Figure 2b shows computed BOD concentrations for the year 2050 resulting from projected changes in urban population (Fig. S9), intensive livestock farming (Figs S10 and S11), and river discharge (Fig. S8), the latter due to climate change and changes in water extractions to support a growing global population. A sensitivity analysis suggests that the effect of temperature change on natural degradation is secondary (see section S1) and it is thus not included. In all scenarios, wastewater treatment rates are kept constant at their current levels. As such, computed results give an indication of pollution impacts in the absence of additional investments beyond current treatment capacity to curb the global sanitation crisis.

Figure 2 shows that by 2050 the biggest deterioration is projected to occur in India, sub-Saharan Africa and Mexico, with many smaller regions all over the world also facing substantial challenges. Urbanization is the main factor in Africa, India, China, and parts of South America (refs 18 and 19; Figs S9 and S14a). Intensification of livestock farming is mainly a factor in India, Africa and South America (Fig. S14b), while intensive livestock farming in Europe and China is expected to stay constant or decline in the coming decades, thereby reducing impacts on river basins in these regions. Finally, regions with a significant decrease in discharge, such as most parts of Europe, West Africa, western and southern Asia, and Latin America excluding the Amazon, experience a decrease in dilution capacity, which translates into increased pollution levels in several river basins (Fig. S14c: Ganges, Yangtze, Indus, Parana, Nile, Danube, Niger). Other basins (Yellow & Huai, Mississippi) benefit from an increased dilution capacity due to a projected increase in mean discharge (Fig. S8). However, increased discharge may also have negative effects not accounted for in our analysis. For example, cities may experience pollution surges as increases in urban stormwater overload the capacity of sewer systems and wastewater treatment plants^20^.

In terms of number of people affected, except for the Rhine, all basins listed in Fig. 3 are projected to experience increasing impacts in 2050 compared to 2000, with the largest increases in Ganges, Indus, Nile, and Niger. Reasons for these increases differ by basin. Urban population growth for the Yellow and Huai, Yangtze, and Mississippi, decreases in river discharge for the Danube basin, and a combination of all three factors for the Ganges, Indus, Parana, Nile, and Niger basins. Globally, a total of 2.5 billion people (26% of global population) will be affected by living near polluted rivers (BOD >5 mg/l) in the year 2050, up from 1.1 billion people (19%) in the year 2000. Basins listed in Fig. 3 account for about half of affected people. This is a conservative estimate because several factors not accounted for in our analysis (industrial pollution, eutrophication, seasonal discharge cycles and smaller streams with limited dilution capacity) would further increase these numbers^4^,^6^.

## Discussion

### Portfolio of wastewater solutions

Given that projected impacts are largest in developing regions, where wastewater treatment is still limited (Fig. S6), there is an urgent and growing need for affordable wastewater solutions. In developed countries, organic river pollution from urban effluents has historically been reduced by a combination of environmental regulation and large-scale wastewater treatment^6^. Such investments are likely not within reach of many developing regions, thus requiring a more decentralized approach^21^,^22^,^23^. Current efforts in that direction include subsidizing adoption of improved sanitation^2^, decentralized low-energy methods of wastewater treatment^24^,^25^, and novel ways of reusing wastewater^8^.

As intensification and growth of livestock farming is driven by the global demand for meat and dairy, pollution impacts may also be tackled by economic policies such as pricing to more accurately reflect environmental and social costs. This is especially important in cases where international trade spatially separates meat consumption from the negative impacts of its production^26^. Such pricing policies, combined with a focus on raising environmental awareness of consumers, are useful tools for reducing global demand for meat and dairy.

Finally, additional pressure in several regions of projected reductions in river dilution capacity ties pollution control to basin-scale water management^16^,^27^ and climate adaptation^20^,^28^, making dialogues with provincial and national governments as well as international cooperation important^29^. Any measures that increase freshwater availability may help here, such as reservoir releases and conjunctive use. Making water available for pollution control competes with several other water users and thus a proper balance needs to be found that considers all stakeholders.

In short, coordinated efforts are needed that combine innovative and affordable wastewater treatment with integrated water management, targeted economic policies, and consumer education. Spatially explicit evaluation of the full range of options holds great promise in addressing impacts of the global sanitation crisis, yet it remains largely unexplored.

## Methods

### Modelling strategy and equations

Any model must consider the trade-off between model complexity and data availability^30^. As such, a global model of organic river pollution cannot be expected to include all details and processes. Instead, our modelling strategy was to focus on the main drivers affecting spatial patterns of organic pollution in global river networks. Favourable comparison of the resulting model predictions to data (Fig. S11 and Table S9 in the Supplement) confirms the validity of this approach. Assumptions and possible extensions of our model in light of available data are discussed in the next section. Here, we first provide more details on the conceptual and mathematical underpinnings of the model. Additional details on the methodology are available in the Supplement to this article.

**Figure 5:**
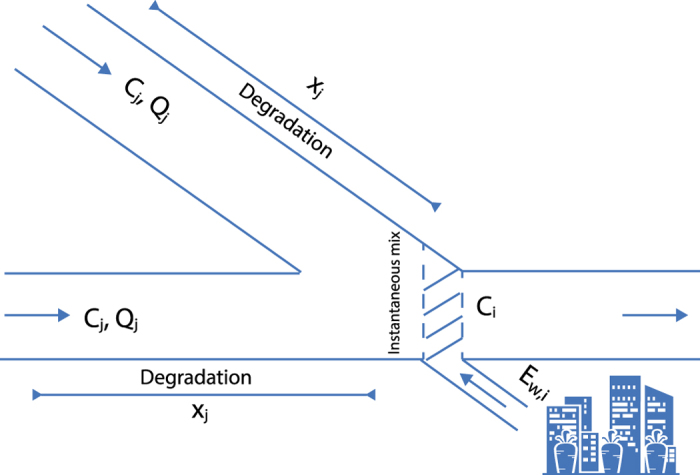
Conceptual diagram of computing in-stream BOD concentrations.

Figure 5 shows a conceptual diagram of our approach for computing in-stream BOD concentrations as a function of urban organic pollution production, wastewater treatment, intensive livestock farming, upstream-downstream transport, dilution, and natural degradation. The approach follows Voβ *et al*.^11^, who applied a similar model to European river networks. Vörösmarty *et al*.^4^ also included BOD in their global analysis but did not account for natural degradation, neither did they look at future changes. Our calculation is implemented on a 0.5-degree grid, and thus only major rivers are taken into account, with urban regions and intensive farming areas that are within 5 km of a major stream included as point sources.

Estimating in-stream BOD concentrations along discretized river networks is based on a local mass balance that relates downstream concentration in a river segment or grid cell to concentrations in upstream segments: C_i_ is BOD concentration in segment i (mg/l) after mixing, Q_i_ is discharge in segment i (l/day), x_j_ is length of river segment j (m), E_W, i_ is local BOD load from urban wastewater and intensive livestock farming into segment i (mg/day), calculated as:


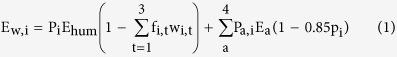


where P_i_ is urban population in grid cell *i, E*_*hum*_ is country-average BOD production from urban population (mg/person/day), f_i, t_ is the fraction of urban domestic wastewater collected for treatment type t with treatment efficiency *w*_*i, t*_, P_a, i_ is the population of livestock type a raised in intensive farming system in grid cell i (a = chicken, pig, water buffalo and cattle), E_a_ is average BOD production from livestock type a (mg/stock/day), p_i_ is the proportion of livestock farming wastewater collected for treatment. There were no data available for intensive livestock farming treatment levels, all livestock farming treatment levels are assumed as secondary, i.e. 85% treatment efficiency^31^. Our approach differs from previous work where BOD loads were based on estimated nitrogen (N) emissions and BOD:N ratios^4^.

Assuming stream and wastewater discharge are at steady state, and instantaneous full mixing of all flows, the total BOD load L_BOD, i_ into downstream segment i can be calculated as:


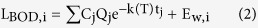


where the sum is over all upstream river segments draining into grid cell i. The instantaneous mixing concentration of BOD is:


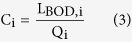


The travel time for BOD in each upstream segment is calculated as:


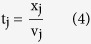


where v_j_ is average flow velocity in river segment j (m/day).

The first-order degradation rate coefficient k is temperature T dependent according to^32^:


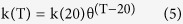


where typical values for θ range from 1.02 to 1.15, with a value of 1.047 used in many models^11^,^33^. The reported range for laboratory-measured k values is from 0.3 to 0.5 day^−1^ at a temperature of 20 °C, which is considered representative of field conditions^34^,^35^. A value of 0.35 day^−1^ was used in our model, somewhat higher (more conservative) than the value of 0.23 day^−1^ used in a previous study^11^.

Calculations for the year 2050 are based on mean projected urban population, intensive livestock farming and discharge, derived from an ensemble of two IPCC emission scenarios (A2 - fast growth, and B1 - slow growth), three coupled atmosphere-ocean General Circulation Models (GCMs), and one Global Hydrological Model (GHM), i.e. WaterGAP^36^. Projections suggest that air temperature will increase by about 1 °C in 2050, relative to the 1986–2005 period^37^. A sensitivity analysis showed that an increase in air temperature of 2 °C would lead to an increase in annual average river temperature of 1.3 °C^38^. A worldwide projected increase of average first-order decay rates due to global warming is up to 10%^39^, a small change, suggesting that the direct temperature effect of climate change on river BOD concentrations is small, especially compared to effects of changes in river discharge.

### Assumptions and possible extensions of the model

Here, we discuss the rationale behind several model assumptions, and outline possible model extensions in light of available data. A first group of assumptions relates to sources of organic river pollution that are not explicitly included in the model:

- industrial sources: a previous continental-scale study in Europe^31^ concluded that organic loads from domestic and livestock farming sources are each at least ten times greater than contributions from industrial activities. As such, organic pollution from industry is considered a secondary driver and not included in the model. However, locally, industrial pollution may still be an important factor: areas where our model underestimates observed concentrations due to potential industrial activities are identified and discussed in the Supplement. In the absence of globally extensive datasets, efforts to add industrial sources to the model should focus on these areas first.
- agricultural non-point sources: by their very nature, non-point sources, such as extensive livestock farming and manure applied to agricultural fields, contribute much lower BOD values than effluents from intensive livestock farming, albeit over larger areas. Previous work^13^ in extensive livestock areas suggests that in-stream BOD levels exceed the range of natural water only during dry periods. Our model is limited to long-term average conditions (steady-state) and ignores such seasonal or shorter-term effects on organic pollution.
- rural domestic sources: following other global river pollution studies^11^,^40^,^41^, we assumed that organic pollutants from rural areas do not enter rivers due to either collection of human waste in latrines and septic tanks, or retention and degradation in soil.
- wastewater interception and diversion: local effects of urban pollution interception and diversion (e.g. to the ocean as in the San Francisco Bay Area)^42^,^43^ are not included but could be added where available.

A second group of assumptions relates to parameterization of pollution and degradation processes:

- BOD degradation rates: as mentioned earlier, a constant rate coefficient k of 0.35 day^−1^ is used. This value is similar to laboratory measured values and to a value of 0.23 day^−1^ used in another large-scale modelling study^11^. Previous work^34^,^35^ has considered these values representative of rivers with discharge larger than 22.7 m^3^/s^32^ (i.e. most of the rivers in our study). While k values may change spatially with river hydraulic conditions, these effects are currently not included in the model. A possible model extension is to include these effects via settling and bed effects equations in shallow streams^34^. We note that the direct effect of river flow velocity on degradation *is* included (Eq. 4). In addition, neither secondary effects of organic pollution such as eutrophication nor light degradation in in-stream reservoirs are included in our calculation^4^, as it depends on daily or seasonal variation and oxidizable nitrogen compounds in polluted waters^44^,^45^.
- wastewater treatment fractions and efficiencies: assumed spatial distributions of treatment fractions reflect available data (by city or country for domestic sources, and by region for livestock farming; see section S1.2 and Table S1 in the Supplement). Similarly, domestic treatment efficiencies are only available by country. In the absence of systematic data on treatment efficiency in intensive livestock farming, we assumed a uniform efficiency of 85% (secondary level) based on the following considerations:(i) other studies^46^,^47^ considered intensive livestock farming a manufacturing activity subject to secondary or tertiary treatment, at least in European countries^31^, (ii) in Asia, effluents from large livestock farms are either diluted and reused for irrigation, or processed through (an) aerobic treatment plants such as lagoons, resulting in organic removal rates that approach secondary treatment levels^48^,^49^, and (iii) a sensitivity analysis reveals that computed BOD concentrations are relatively insensitive to the assumed efficiency in Africa and South America because of the low fractions of livestock farming wastewater treatment in these regions (from 6% to 20%, Fig. S6).

Finally, as with other climate change studies, our projections are subject to uncertainties in future population (e.g. grid-scale projected changes in urban population assume uniform exponential growth rates across each country^50^) and river discharge as simulated by a limited number of scenarios and generally imperfect global climate-hydrological models^51^. We note that projected river discharge applied in our model generally agrees with forecasting from IPCC (see Fig. S2). In addition, one aim of our study is to estimate consequences of river pollution in the absence of additional investments in wastewater treatment. Thus, our projected results rely on the assumption that wastewater treatment remains at current levels.

## Additional Information

**How to cite this article:** Wen, Y. *et al*. Organic pollution of rivers: Combined threats of urbanization, livestock farming and global climate change. *Sci. Rep.* **7**, 43289; doi: 10.1038/srep43289 (2017).

**Publisher's note:** Springer Nature remains neutral with regard to jurisdictional claims in published maps and institutional affiliations.

## Supplementary Material

Supplementary Materials

## Figures and Tables

**Figure 1:**
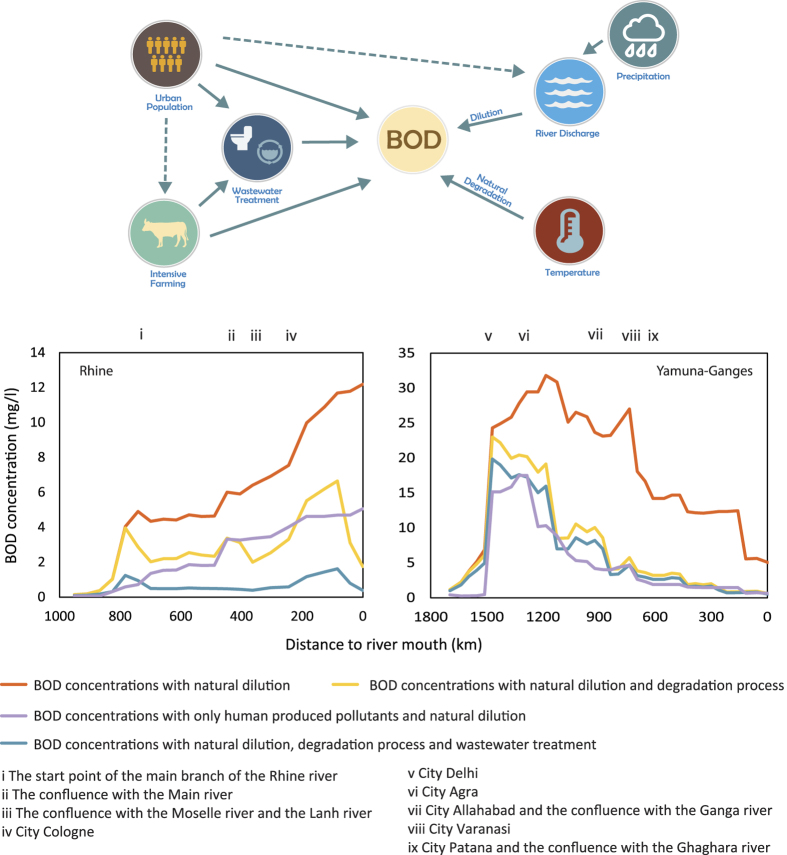
(**a**) Variables and processes affecting organic pollution of rivers, expressed as in-stream Biochemical Oxygen Demand (BOD); (**b**) Calculated in-stream BOD concentration profiles from headwater to river mouth for the year 2000 along the main stem of the Rhine and Yamuna-Ganges rivers. Without natural degradation and wastewater treatment (purple profiles), BOD concentrations gradually increase along the densely populated Rhine, whereas for the Yamuna-Ganges river a rapid increase near the cities of Delhi and Agra is followed by dilution with freshwater from several large tributaries. Pollutant loading from livestock farming adds significant pollution in both basins (red profiles). When natural degradation is taken into account (yellow profiles), BOD concentrations decrease significantly in both rivers, illustrating the self-cleaning capacity of natural rivers^12^. Wastewater treatment (blue profiles) further reduces BOD concentrations, especially in the Rhine, but also near Delhi and Agra, which have higher rates of wastewater treatment than smaller cities further downstream^52^.

## References

[b1] Pruss A., Kay D., Fewtrell L. & Bartram J. Estimating the Burden of Disease from Water, Sanitation, Hygene at a Global Level. Environ. Health Perspect. 110, 537 (2002). 12003760 10.1289/ehp.110-1240845 PMC1240845

[b2] UNICEF & WHO. Progress on Sanitation and Drinking Water: 2015 Update and MDG Assessment. (UNICEF, 2015).

[b3] Sirota J., Baiser B., Gotelli N. J. & Ellison A. M. Organic-matter loading determines regime shifts and alternative states in an aquatic ecosystem. Proc. Natl. Acad. Sci. USA. 110, 7742–7 (2013). 23613583 10.1073/pnas.1221037110 PMC3651476

[b4] Vörösmarty C. J. . Global threats to human water security and river biodiversity. Nature 467, 555–561 (2010). 20882010 10.1038/nature09440

[b5] Malaj E. . Organic chemicals jeopardize the health of freshwater ecosystems on the continental scale. Proc. Natl. Acad. Sci. 111, 9549–9554 (2014). 24979762 10.1073/pnas.1321082111 PMC4084479

[b6] Meybeck M. Global analysis of river systems: from Earth system controls to Anthropocene syndromes. Philos. Trans. R. Soc. Lond. B. Biol. Sci. 358, 1935–55 (2003). 14728790 10.1098/rstb.2003.1379 PMC1693284

[b7] Bruinsma J. World agriculture: towards 2015/2030: an FAO perspective. FAO (Earthscan Publications Ltd, 2003).

[b8] Nelson K. L. & Murray A. Sanitation for Unserved Populations: Technologies, Implementation Challenges, and Opportunities. Annu. Rev. Environ. Resour. 33, 119–151 (2008).

[b9] McDonald R. I. . Urban growth, climate change, and freshwater availability. Proc. Natl. Acad. Sci. USA. 108, 6312–6317 (2011). 21444797 10.1073/pnas.1011615108 PMC3076880

[b10] Milly P. C. D., Dunne K. A. & Vecchia A. V. Global pattern of trends in streamflow and water availability in a changing climate. Nature 438, 347–350 (2005). 16292308 10.1038/nature04312

[b11] Voß A. . Continental scale modelling of in-stream river water quality: a report on methodology, test runs, and scenario application. Hydrol. Process. 26, 2370–2384 (2012).

[b12] Streeter H. W. & Phelps E. B. A study of the pollution and natural purification of the Ohio River. (US Department of Health, Education, & Welfare, 1958).

[b13] Hooda P. S., Edwards A. C., Anderson H. A. & Miller A. A review of water quality concerns in livestock farming areas. Sci. Total Environ. 250, 143–167 (2000). 10811258 10.1016/s0048-9697(00)00373-9

[b14] Dinda S. Environmental Kuznets Curve Hypothesis: A Survey. Ecol. Econ. 49, 431–455 (2004).

[b15] Daniel R. & Vining J. The growth of core regions in the third world. Sci. Am. 252, 42–49 (1985).

[b16] UNESCO-WWAP. *Water for people, water for life*. (UNESCO, 2003).

[b17] Ministry of Housing and Urban-Rural Development. *China Urban Waste Water Collection and Treatment Status Report 2000–2004*. (Ministry of Housing and Urban-Rural Development of China, 2005).

[b18] United Nations. World Urbanization Prospects The 2014 Revision. (United Nations, 2014).

[b19] Cohen J. E. Human Population: The Next Half Century. Science (80-.). 302, 1172–1175 (2003). 10.1126/science.1088665 14615528

[b20] IPCC. Climate change 2014: Impact, adaption and vulnerability. (Cambridge University Press, 2014).

[b21] Narain S. Sanitation for all. Nature 486, 185–185 (2010). 10.1038/486185a 22699595

[b22] Guiteras R., Levinsohn J. & Mobarak A. M. Encouraging sanitation investment in the developing world: A cluster-randomized trial. Science (80-.). 348, 903–906 (2015). 10.1126/science.aaa0491 25883316

[b23] Kaiser J. For toilets, money matters. Science (80-.). 348, 272 (2015). 10.1126/science.348.6232.272 25883335

[b24] Massoud M. A., Tarhini A. & Nasr J. A. Decentralized approaches to wastewater treatment and management: Applicability in developing countries. J. Environ. Manage. 90, 652–659 (2009). 18701206 10.1016/j.jenvman.2008.07.001

[b25] WHO. Reinventing the toilet for 2.5 billion in need. Bull. World Health Organ. 92, 465–544 (2014). 10.2471/BLT.14.020714 PMC4121873 25110370

[b26] Burke M., Oleson K., McCullough E. & Gaskell J. A global model tracking water, nitrogen, and land inputs and Virtual transfers from industrialized meat production and trade. Environ. Model. Assess. 14, 179–193 (2009).

[b27] C. Rosenzweig W. D., Solecki S. A. & Hammer S. M. Climate change and cities: first assessment report of the Urban Climate Change Research Network. (Cambridge University Press, 2011).

[b28] Bloomberg M. R., Sachs J. D. & Small G. M. Climate Change Adaptation in New York City: Building a Risk Management Response. Ann. N. Y. Acad. Sci. 1196, 1–3 (2010). 10.1111/j.1749-6632.2009.05415.x 21198660

[b29] Rosenzweig C., Solecki W., Hammer S. A. & Mehrotra S. Cities lead the way in climate–change action. Nature 467, 909–911 (2010). 20962822 10.1038/467909a

[b30] Schoups G., van de Giesen N. C. & Savenije H. H. G. Model complexity control for hydrologic prediction. Water Resour. Res. 44, n/a-n/a (2008).

[b31] Williams R. . Assessment of current water pollution loads in Europe: Estimation of gridded loads for use in global water quality models. Hydrol. Process. 26, 2395–2410 (2012).

[b32] Thomann R. V. & Mueller J. A. Principles of surface water quality modeling and control. (Waveland press, 1987).

[b33] Jolankai G. Basic river water quality models. UNESCO (UNESCO, 1997).

[b34] Chapra S. C. Surface water quality modeling. (waveland Pr Inc, 1997).

[b35] Raymond M. W. & Archie J. M. In-Stream Deoxygenation Rate Prediction. J. Environ. Eng. Div. 105, 323–335 (1979).

[b36] Chen C., Hagemann S., Clark D., Folwell S., Gosling S., Haddeland I., Hanasaki N., Heinke J., Ludwig F., Voβ F. & Wiltshire A. Projected hydrological changes in the 21st century and related uncertainties obtained from a multi-model ensemble. (EU WATCH water and global change, 2011).

[b37] IPCC. Climate Change 2014: Synthesis Report. (IPCC, 2014).

[b38] van Vliet M. T. H., Ludwig F., Zwolsman J. J. G., Weedon G. P. & Kabat P. Global river temperatures and sensitivity to atmospheric warming and changes in river flow. Water Resour. Res. 47, 1–19 (2011).

[b39] Punzet M., Voß F., Voß A., Kynast E. & Bärlund I. A Global Approach to Assess the Potential Impact of Climate Change on Stream Water Temperatures and Related In-Stream First-Order Decay Rates. J. Hydrometeorol. 13, 1052–1065 (2012).

[b40] Van Drecht G., Bouwman a. F., Harrison J. & Knoop J. M. Global nitrogen and phosphate in urban wastewater for the period 1970 to 2050. Global Biogeochem. Cycles 23 (2009).

[b41] Mayorga E. . Global Nutrient Export from WaterSheds 2 (NEWS 2): Model development and implementation. Environ. Model. Softw. 25, 837–853 (2010).

[b42] Riviere J. W. M. la. Threats to the World’s Water. Sci. Am. 261 (1989).

[b43] Council N. R. Academia Nacional de la Investigación Científica, A. C. & Academia Nacional de Ingeniería, A. Mexico City’s Water Supply. (National Academies Press, 1995), doi: 10.17226/4937.

[b44] COX B. A review of dissolved oxygen modelling techniques for lowland rivers. Sci. Total Environ. 314–316, 303–334 (2003). 10.1016/s0048-9697(03)00062-7 14499539

[b45] Texas water development board. simulation of water quality in streams and canals (1971).

[b46] Flörke M. . Domestic and industrial water uses of the past 60 years as a mirror of socio-economic development: A global simulation study. Glob. Environ. Chang. 23, 144–156 (2013).

[b47] Department of Economic and Social Affairs Statistics Division, U. N. *International Standard Industrial Classification of All Economic Activities, Rev.3.1*. (United Nations, 2008).

[b48] Ju X., Zhang F., Bao X., Römheld V. & Roelcke M. Utilization and management of organic wastes in Chinese agriculture : Past, present and perspectives. Sci. China Ser. C Life Sci. 48, 965–979 (2005). 16512218

[b49] IAEA. GuidGuidelines for Sustainable Manure Management in Asian Livestock Production Systems (2008).

[b50] Balk D., Yetman G. & Sherbinin A. De. Construction of gridded population and poverty data sets from different data sources. In European Forum for Geostatistics Conference 5–7 (2010).

[b51] Weaver A. J. & Zwiers F. W. Uncertainty in climate change. Nature 407, 571–572 (2002). 10.1038/35036659 11034189

[b52] Raghupathi U. P. Status of Water Supply,Sanitaion and Solid Waste Management in Urban Areas. (CPHEEO, 2005).

[b53] Esri. ArcMap 10.1.

[b54] MathWorks. MatLab R2012b.

