# Supplementary material for: Organic pollution of rivers: Combined threats of urbanization, livestock farming and global climate change
Source: Sci Rep. 2017 Feb 23;7:43289. doi: 10.1038/srep43289 (PMC5322379; doi:10.1038/srep43289)
Supplement: Supplementary Materials [file srep43289-s1.pdf]

Supplementary Materials for

Organic pollution of rivers: combined threats of urbanization, livestock farming and global climate change

Yingrong Wen*, Gerrit Schoups, Nick van de Giesen

*Correspondence to: Y.Wen@tudelft.nl

This document contains information on (1) all spatially distributed model inputs, and (2) simulated BOD concentrations for historical and future conditions under various scenarios, including a comparison to observed historical BOD concentrations.

1 Model inputs

Data input into the model equations can be divided into model inputs for historical calculations (year 2000) and for changes in the future (year 2050). Table S1 gives an overview of all data sources and methods related to estimating model inputs.

1.1 Model inputs for historical calculations (year 2000)

The river network was derived from a global drainage direction map (DDM 30)[1]. DDM30 is a raster map which describes the drainage directions of surface water with a spatial resolution of 0.5 degree. The river length was calculated from DDM 30. Average gridded discharge (from year 1991 to 2000) values were calculated by WaterGAP taken from WATCH 21st century model output[2]. River discharge takes human impacts like dams and water withdrawals and use into account. In the calculation, we only considered the potential active river networks where runoff exceeds 3 mm/yr[3]. The average river discharge in year 2000 is shown in Figure S1. The average river flow velocity was calculated following Schulze et al., 2005[4], which used a simple function of discharge. All calculations are performed on the 0.5-degree grid.

Urban daily per capita BOD loadings depend on diet, metabolism, body weight, food preparation habits, bathing, cleaning and laundering. Table S2 lists average BOD loadings for domestic wastewater in selected countries and regions. The full list of country-specific BOD generation data is available from the US EPA[5]. For countries where BOD generation data is not known, average continent-based data was used. The global distribution of urban population in the year 2000 is shown in Figure S2.

BOD pollution from livestock farming varies due to differences in animal type, diet, age, usage, productivity and management[6]. In our model, we used mean BOD values based on livestock manure production and characterization (Table S3). Tropical livestock units (TLU) are used to provide an equivalent estimate of livestock biomass. One TLU is equivalent to 250kg, where one bovine is equivalent to 1 TLU.

Maps of global distribution of intensive cattle/buffalo production systems were built based on threshold densities in different regions (Table S4). For poultry and pig, such existing maps

with a spatial resolution of 0.05 degree were directly used for calculating production. Global distributions of intensive livestock animal production systems in year 2000 are shown in Figure S3 and Figure S4.

1.2 Wastewater treatment

We used country-average data of domestic wastewater treatment systems for most countries, and treatment fractions were derived from percentages of population connected to different treatment types and percentages of population living in urban areas in the year 2000 (see Figure S5). For India, China and Brazil, downscaled data of wastewater treatment was used. The overall values for BOD removal fractions were estimated as a weighted fraction of no treatment (zero efficiency), primary treatment (25% efficiency), secondary treatment (85% efficiency) and higher treatment (99% efficiency)[7].

Cities in India were divided into four classes based on population size. Wastewater treatment data for metropolitan cities, Class I cities and Class II cities was taken from a research report on the status of water supply, sanitation and solid waste management in urban areas in India [8]. For less populated cities (Table S5), we used country-average values.

Wastewater treatment in eastern China and in urban areas is more developed than in western China and township areas, respectively [9]. The eastern part includes the following provinces and cities: Anhui, Beijing, Chongqing, Fujian, Guangdong, Henan, Hubei, Hunan, Jiangsu, Jiangxi, Shandong, Shanghai, Tianjin and Zhejiang. The urban and township areas were derived from global urban settlement points in the year 2000 [10]. Urban wastewater treatment data in China is shown in Table S6.

Brazilian cities were also divided into five classes based on population size (Table S7), where 10% of treated wastewater receives primary treatment and 68% receives secondary treatment [11].

Intensive livestock farming is considered a manufacturing activity [12,13], thus the fraction of manufacturing wastewater treatment data was applied to intensive livestock farming activities (see Fig. S6).

1.3 Model inputs for changes in the future

We calculated the average change of river discharge from three GCMS (CNCM3, ECHAM and IPSL) under scenarios A2 and B1 (Fig. S7). Runoff is notably projected to become less in southern Europe, western Africa, northeast of South America and southern Asia. River discharge is projected to increase in high latitudes, wet tropics, eastern part of the United States and southeast Asia. These changes generally agree with projections from the IPCC[14].

Estimates and projections of the total national population of each country or area were calculated based on the proportion of the population living in urban areas, which is assumed fixed[15]. The urban population in grid cells was derived from global urban settlement points in 2000[10].

Assuming exponential growth, urban population in 2050 is calculated by country-based urban growth rates for two scenarios (A2: high fertility, B1: low fertility), which were applied to

gridded urban populations in the year 2000[15,16]. The national urban population growth rate reads as follows:

$$r_{sc} = \frac{1}{t} ln \frac{u_2 TP_{sc,2}}{u_1 TP_{sc,1}} \quad (1)$$

Where r_{sc} is the national urban population growth rate under scenario sc , $u_{1..2}$ is the proportion of the population living in urban areas in two different time periods, $TP_{sc,1..sc,2}$ is the total national population and t is the number of years between the two time periods.

We computed urban population under scenarios A2 and B1 and calculated the average, then compared the average with the urban population distribution in 2000 (Fig. S8). The most significant increases in urban population occur in China, Africa and the Indian subcontinent. In eastern Europe, some areas are projected to experience a decrease in urban population.

The country-based projections of livestock animal production in 2050 were taken from the output of the International Model for Policy Analysis of Agricultural Commodities and Trade (IMPACT), developed by IFPRI[17]. In this model, livestock production is determined by the livestock's price and the prices of other competing commodities, the prices of intermediate feed inputs, and the number of animals slaughtered for meat production. We assumed that the proportions of livestock raised in both intensive and extensive production systems remain the same in the future. Changes in intensive livestock farming systems from 2000 to 2050 are shown in Figs. S9 and S10. In 2050, the most significant increases of intensive bovine productions are projected to be in India, central Africa and the Caribbean. The intensive production of chicken in China and Europe is projected to decrease, while it is projected to increase in India and Southeast Asia. The intensive production of pig is projected to increase in the United States and Brazil.

2. Simulated river BOD concentrations

2.1 Comparison to observed river BOD concentrations

Measurements of BOD concentrations were taken from the GEMS global river water quality database, the European Waterbase database, the STORET Data Warehouse of the United States, the Central Pollution Control Board of India, the Ministry of Environmental Protection of the People's Republic of China and the National Water Agency of Brazil[18–22]. Measured data in China is reported in pollution classes with corresponding BOD concentration ranges shown in Table S8[22,23]. We calculated mean values/grades of BOD concentrations at over 700 observation spots from 1991 to 2000 and compared these with our calculations.

The results show that for the complete data set, the model gives satisfactory results because most calculated concentrations (94%) are in the same water quality class as observed data (Table S9), with an underestimation of the number of polluted sites for BOD concentrations in the range 5-10 mg/l. We conclude that model calculations do not disagree with available data when interest is in assessing river water quality in terms of the broad BOD concentration categories of Table S9. In Figure S11, which presents the average simulated and measured BOD concentrations at observation stations, we see that underestimation mainly occurs for rivers in Europe and India. A possible reason for these differences is the contribution of industrial BOD pollution, which is not accounted for in our model. The most important industries in terms of organic river pollution are paper and pulp, iron and steel, non-ferrous

metals, miscellaneous manufacturing, industrial chemicals, beverages, food production, and rubber and petroleum production[24]. For example, the lower Ebro river in Spain receives sewage from three paper mills, which are important contributors of intensive organic pollutants[25]. Industrial activities are concentrated in big cities along the river and industrial point sources contribute more than 70% of organic matter[26], which leads to observed BOD concentrations between 5 and 10 mg/l (based on 6 locations along the Ebro river), whereas simulated BOD concentrations are less than 5 mg/l.. Similarly, the polluted zone of the Krishna river in India is likely due to organic inputs from sugar industries[27,28], which results in an underestimation of 2 polluted sites in the Maharashtra region with BOD concentrations in the range 5-10 mg/l.

2.2 Simulated BOD concentrations separated by contributing factors in 2000

Figure S12(a) shows river organic pollution directly affected by pollutants produced by urban residents and natural dilution in year 2000. Rivers in mid-eastern China, northwest Indian sub-continent, Europe, western United States, Morocco, as well as smaller regions in Japan, Korea, Mexico, Caribbean and South America exhibit the most serious pollution.

Figure S12 (b) shows BOD concentrations with pollutants from urban and intensive livestock framing in the year 2000. The impact is overwhelming when compared to Figure S12(a). China, Korea, Japan, the Indian sub-continent, Europe, the United States, Mexico, South America and smaller parts in Africa and Australia show significant deterioration of river water quality. The effect of decreasing river organic pollution is prominent in parts of Brazil, Nigeria, eastern Europe and southeast China, when natural degradation is also taken into account as shown in Figure 12(c). For river systems in Europe, the United States, Japan, Australia and southeast China, organic pollutions are largely controlled by wastewater treatment plants as shown in Figure 12(d), while rivers in the Indian sub-continent, mid-eastern China, Africa, Mexico, Caribbean and South America still exhibit severe pollution.

2.3 Simulated BOD concentrations for different scenarios with three GCM models

By 2050 the world's urban population is projected to increase by 2.5 billion people, with most of this growth taking place in poor countries, particularly in Asia and Africa[15,29]. Using historical river discharge and intensive livestock production, Figure S13(a) shows that population growth is projected to exacerbate river pollution in parts of India, China, Africa, Mexico, Caribbean and South America. Urban population in most of these regions are also projected to grow due to high-fertility rates[30], referring to the global map of urban population change from 2000 to 2050 in Figure S8. Urban population in Europe, Japan and South Korea are not expected to change much in the coming decades, a few cities will even experience population decline[15]. However, several megacities (e.g. Paris, Birmingham, Tokyo and Seoul) with continuously rising populations are projected to experience high environmental pressures.

The accumulated changes of intensive livestock farming deteriorate river water quality in small parts of central Africa, mid-northern India, Caribbean and South America, as shown in Figure S13(b), in comparison with Figure S13(a). These regions are projected to experience increases of intensive livestock farming as shown on the global map of intensive livestock farming change from year 2000 to year 2050 in Figure S9 and S10.

Finally, Figure S13(c) illustrates the combined effects of changes in urban population, intensive livestock farming and river discharge. Rivers in eastern China, southern India, central Africa, Brazil, Mexico and Caribbean are projected to face the double threat of reduced river discharge and increased pollutants loadings.

Figures S14 to S16 present simulated BOD concentrations using three different GCM models (CNCM3, ECHAM and IPSL) in 2050. River organic pollution calculated by IPSL is the severest. Almost every major river in India is projected to be polluted. South America and central Africa will also face significant organic river pollution.

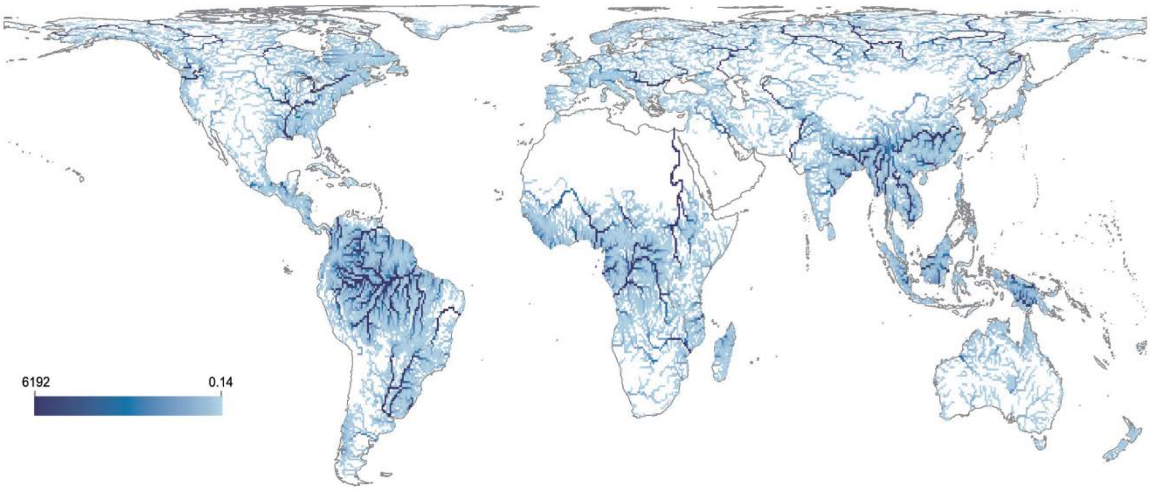

Figure S1. Average river discharge in 2000 (km^3/yr)[31]**.**

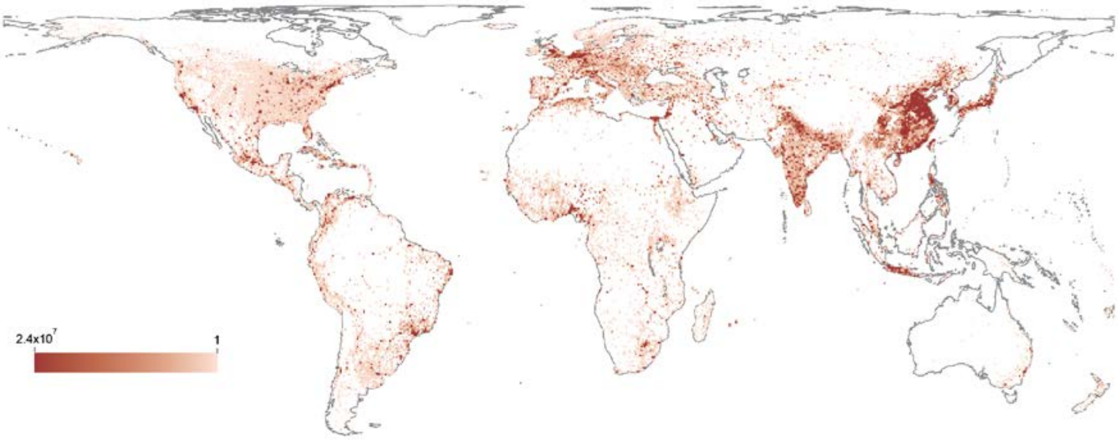

Figure S2. Urban population in 2000 (people per grid cell)[31]**.**

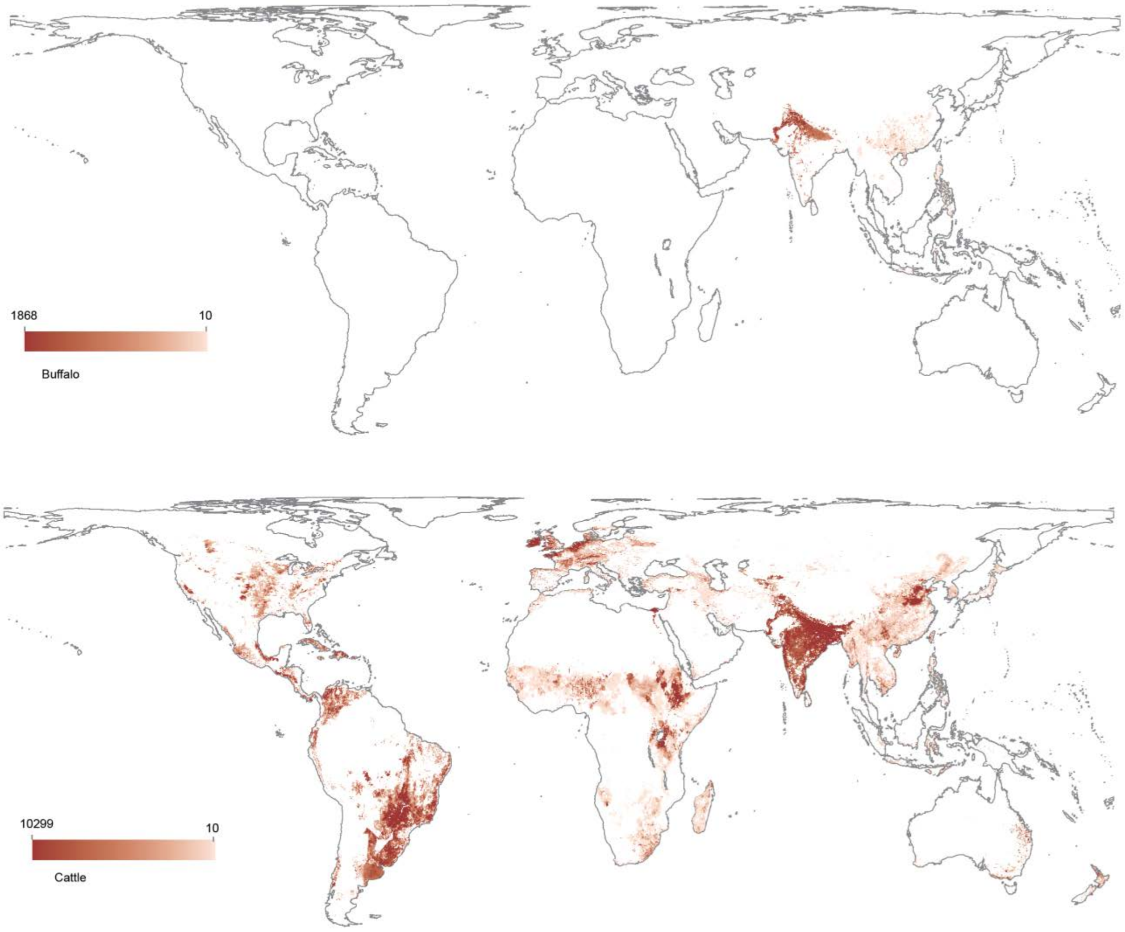

Figure S3. Global distribution of intensive buffalo and cattle production systems in 2000 (stock/km^2)[31].

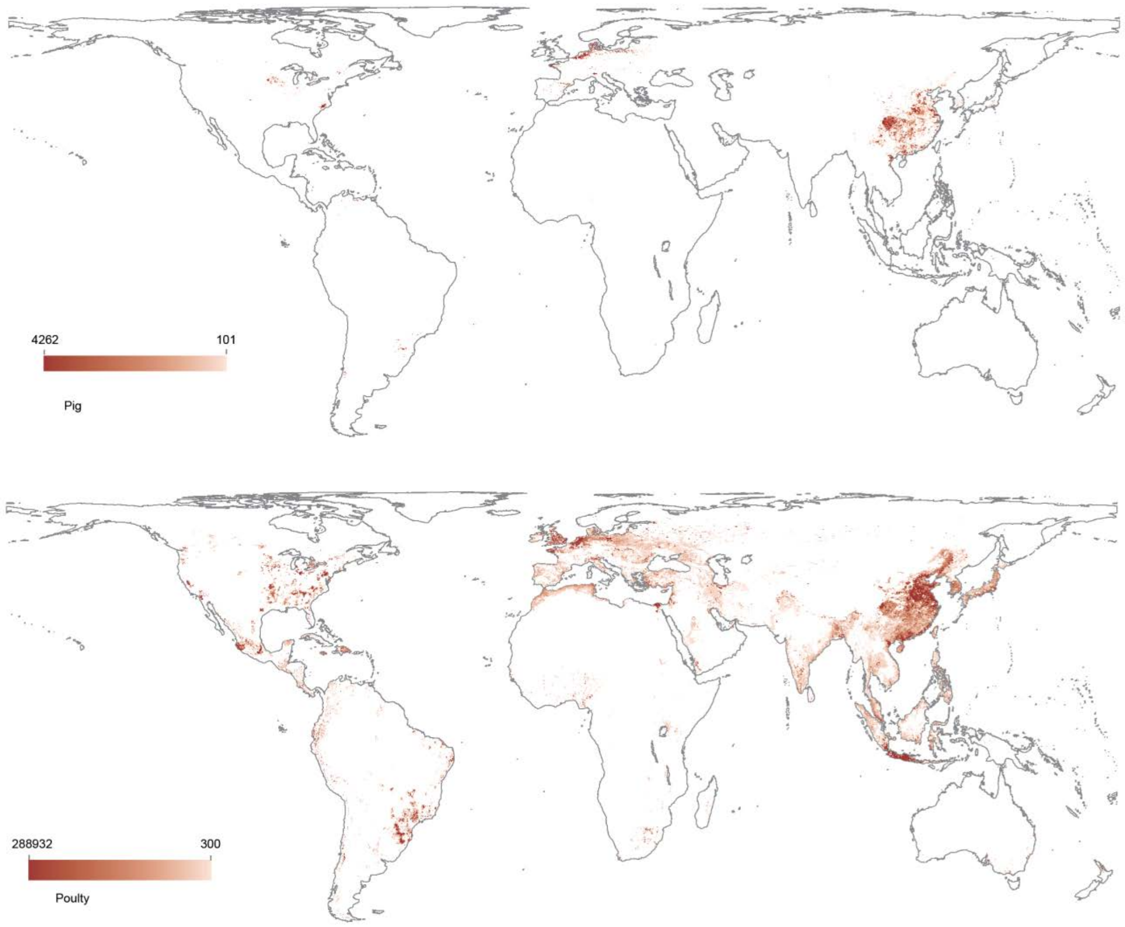

Figure S4. Global distribution of intensive pig and poultry production systems in 2000 (stock/km^2)[31].

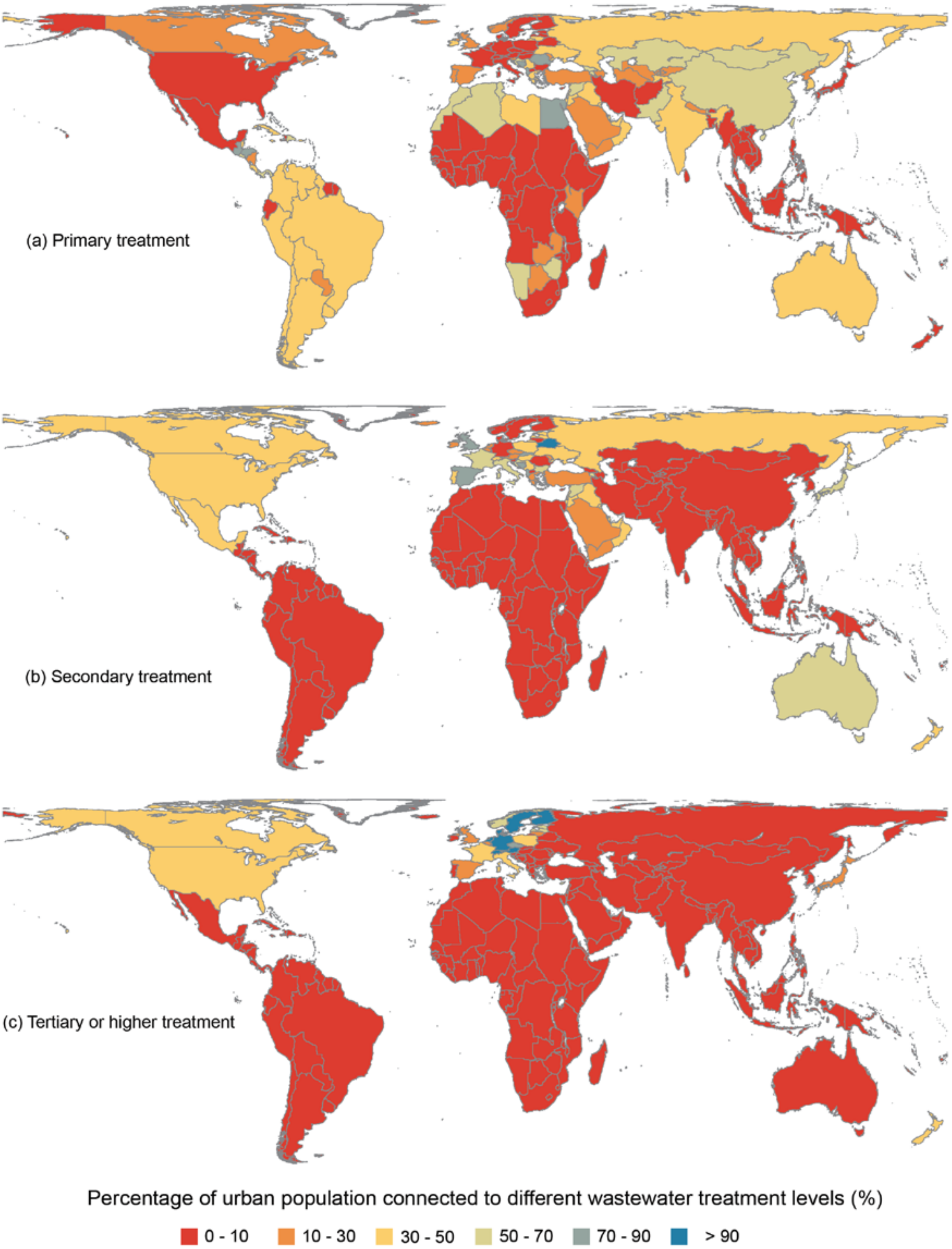

Figure S5. Percentage of urban population connected to different wastewater treatment levels (%)[31].

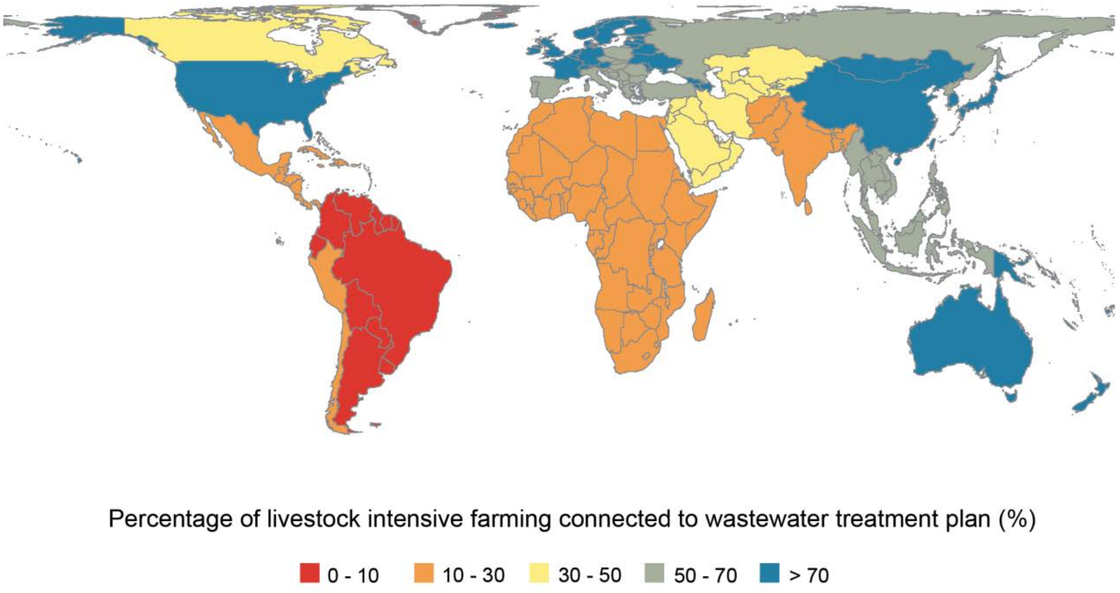

Figure S6. Percentage of intensive livestock farming subject to wastewater treatment (%)[31].

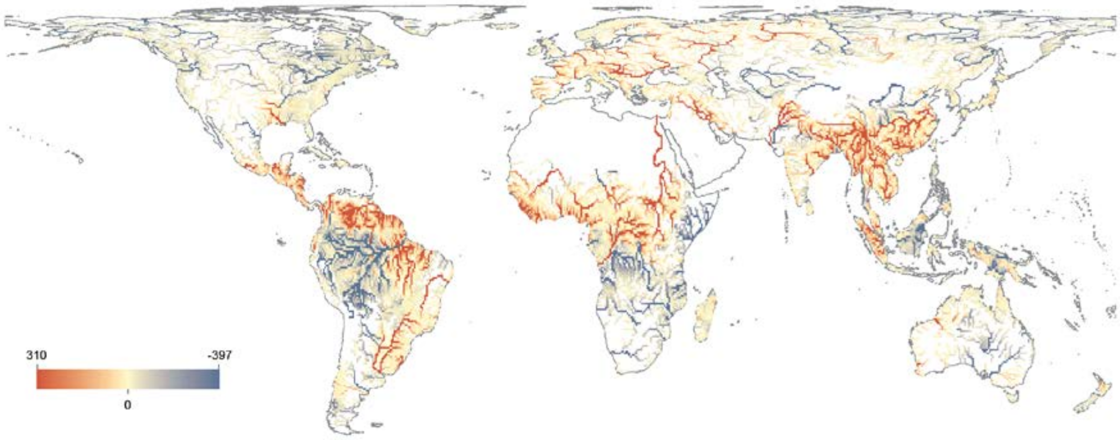

Figure S7. Average change of river discharge from year 2000 to 2050 (km^3/yr)[31].

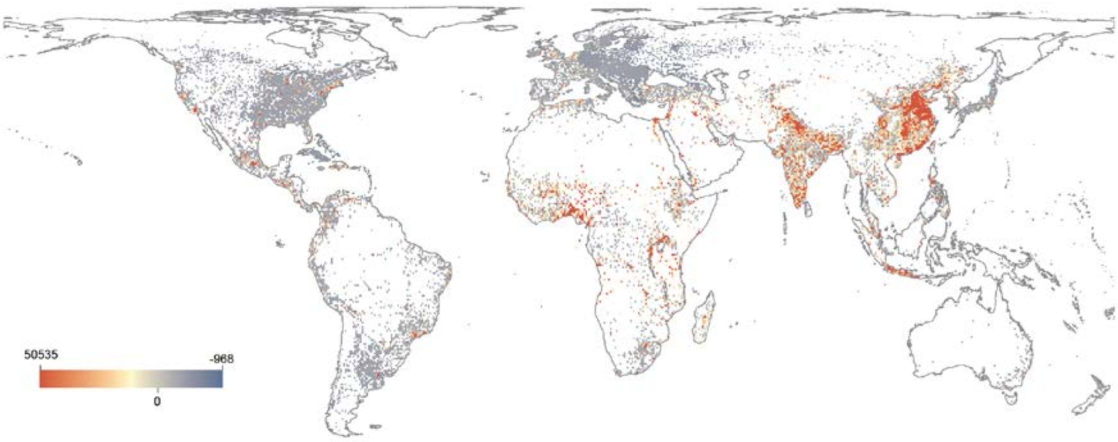

Figure S8. Average change of urban population from 2000 to 2050 (in thousands of people)[31].

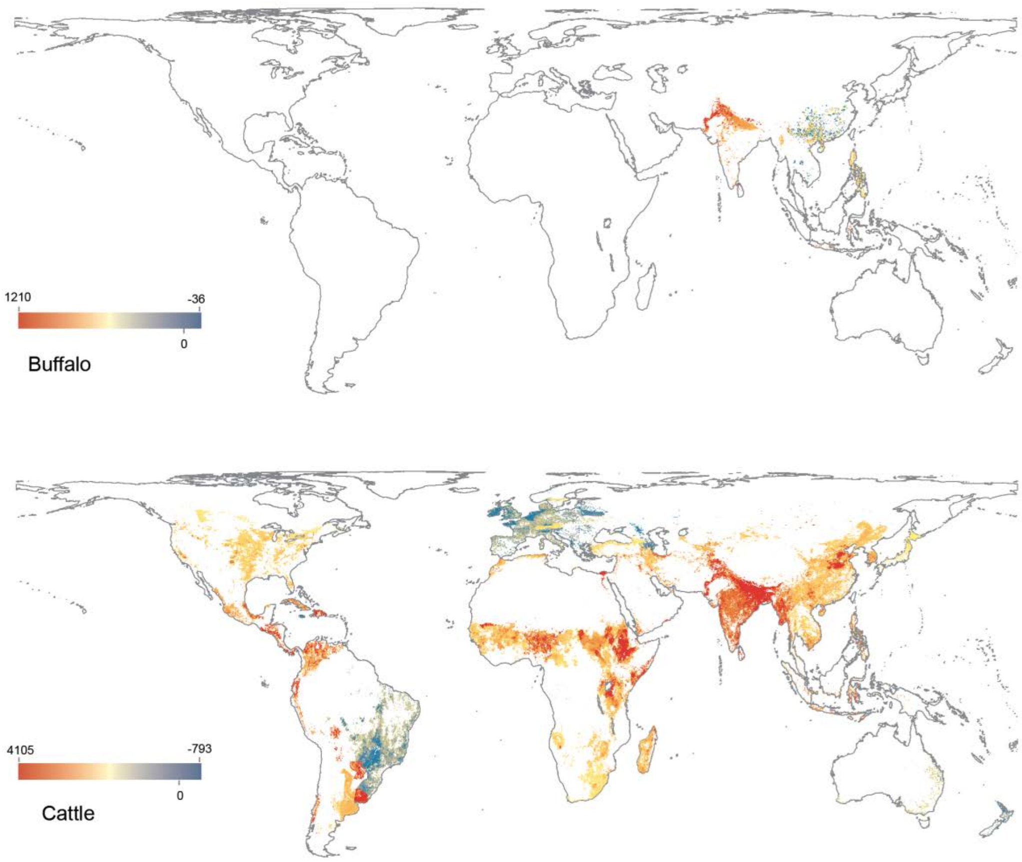

Figure S9. Average change of intensive buffalo and cattle production from 2000 to 2050 (stock/km^2)[31].

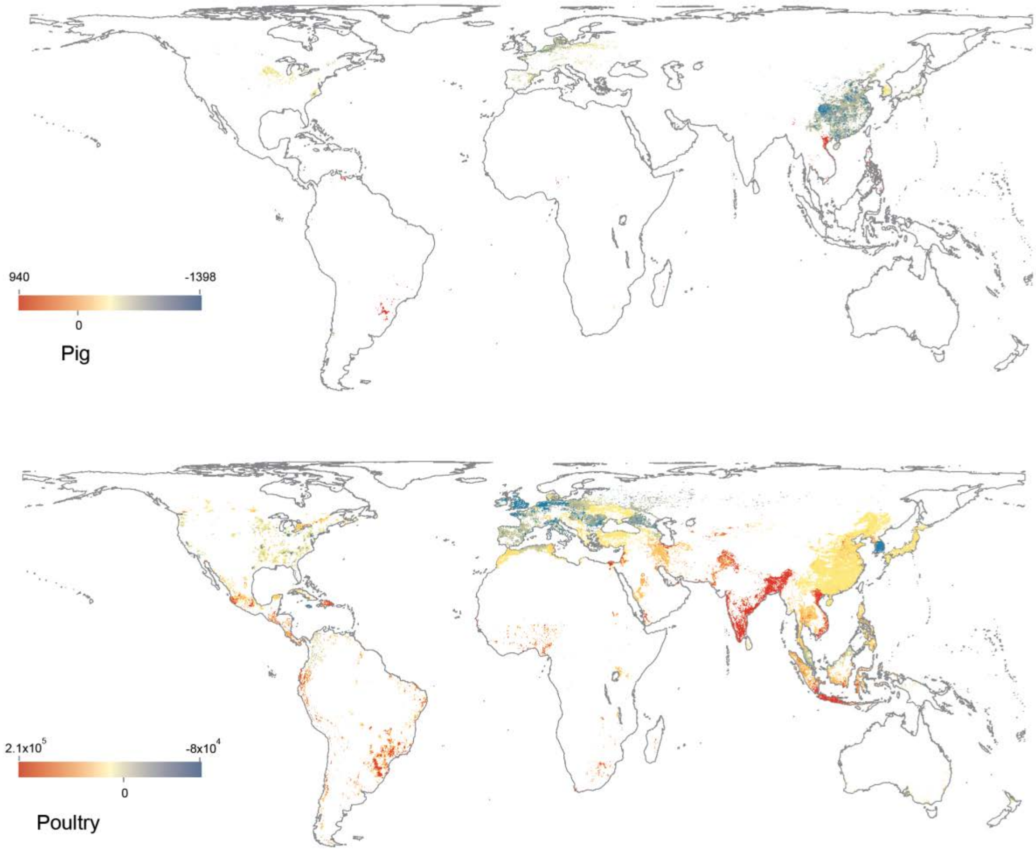

Figure S10. Average change of intensive pig and poultry production from 2000 to 2050 (stock/km^2)[31].

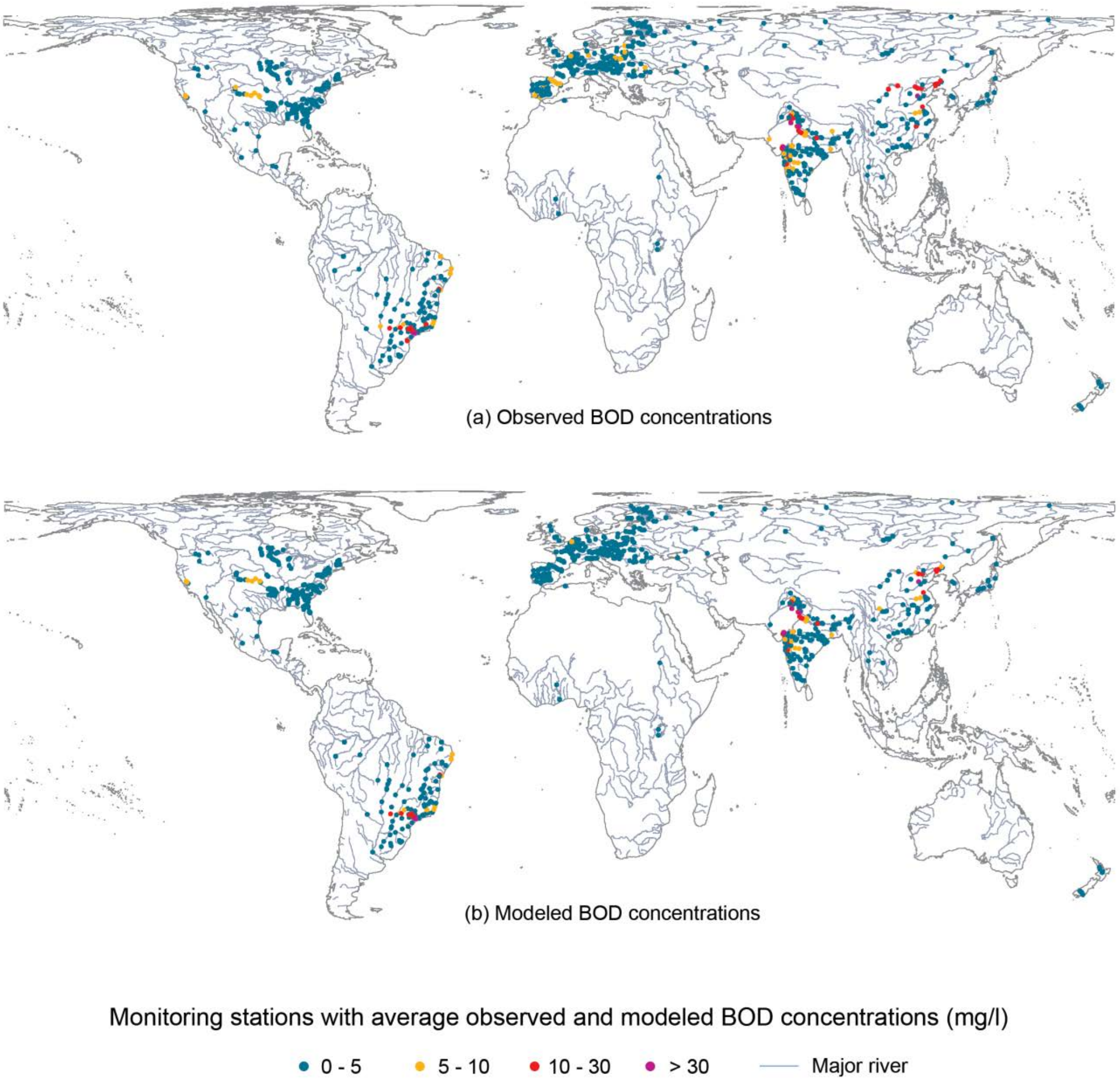

Figure S11. Monitoring stations with average observed and simulated BOD concentration in year 2000[31].

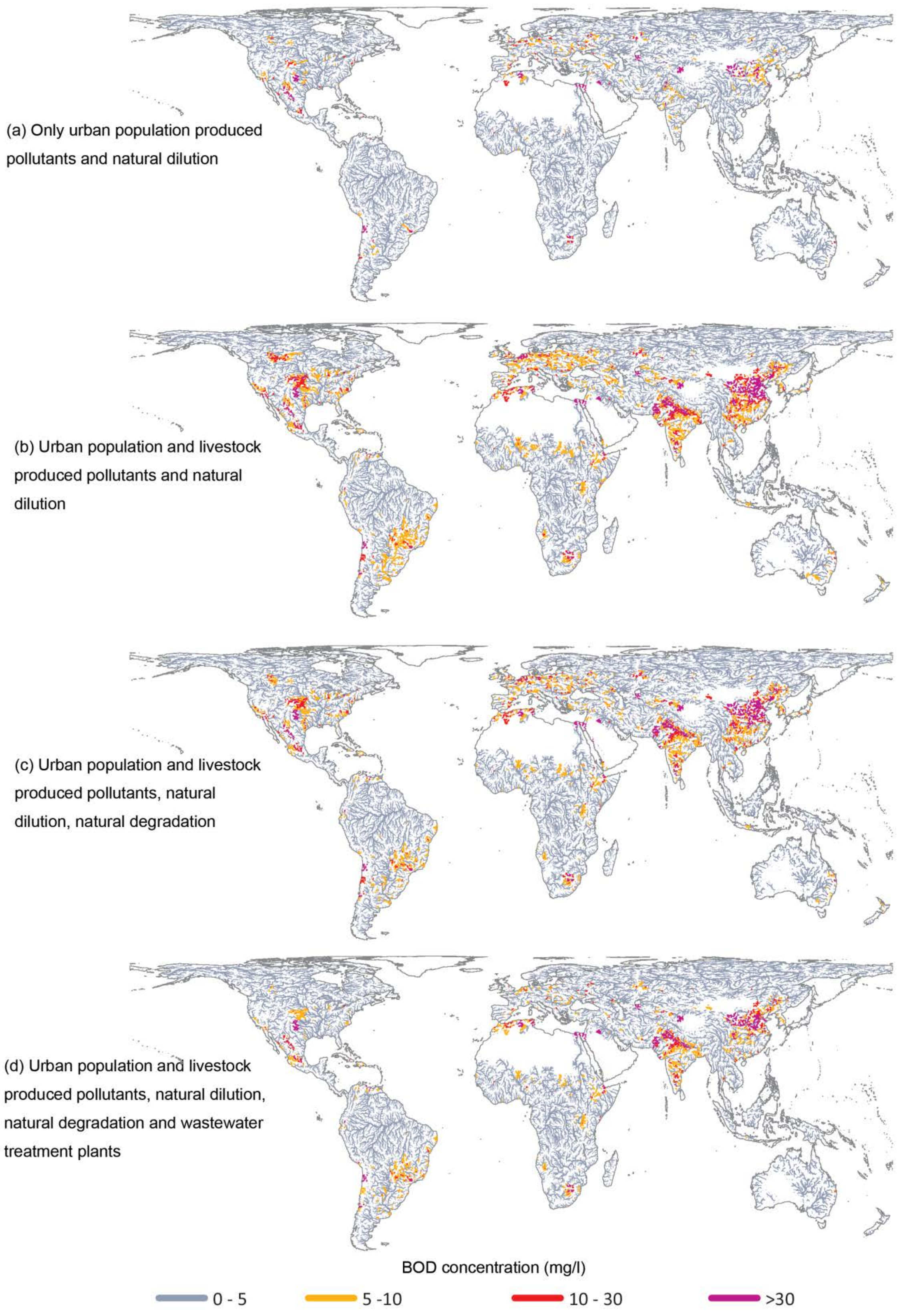

Figure S12. Simulated BOD concentrations in 2000 separated by separated by contributing factors[31,32].

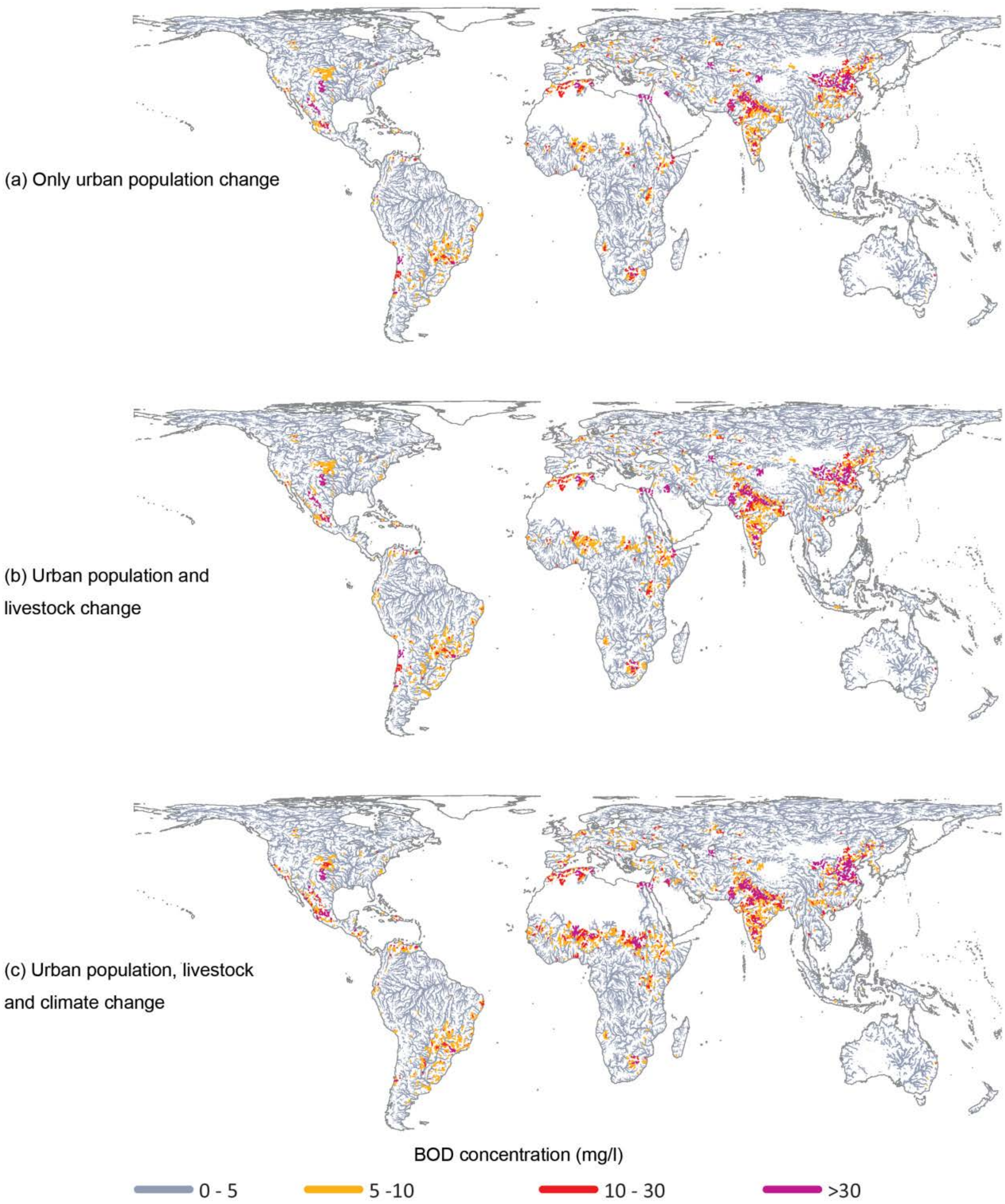

Figure S13. Simulated BOD concentrations in 2050 separated by contributing factors[31,32].

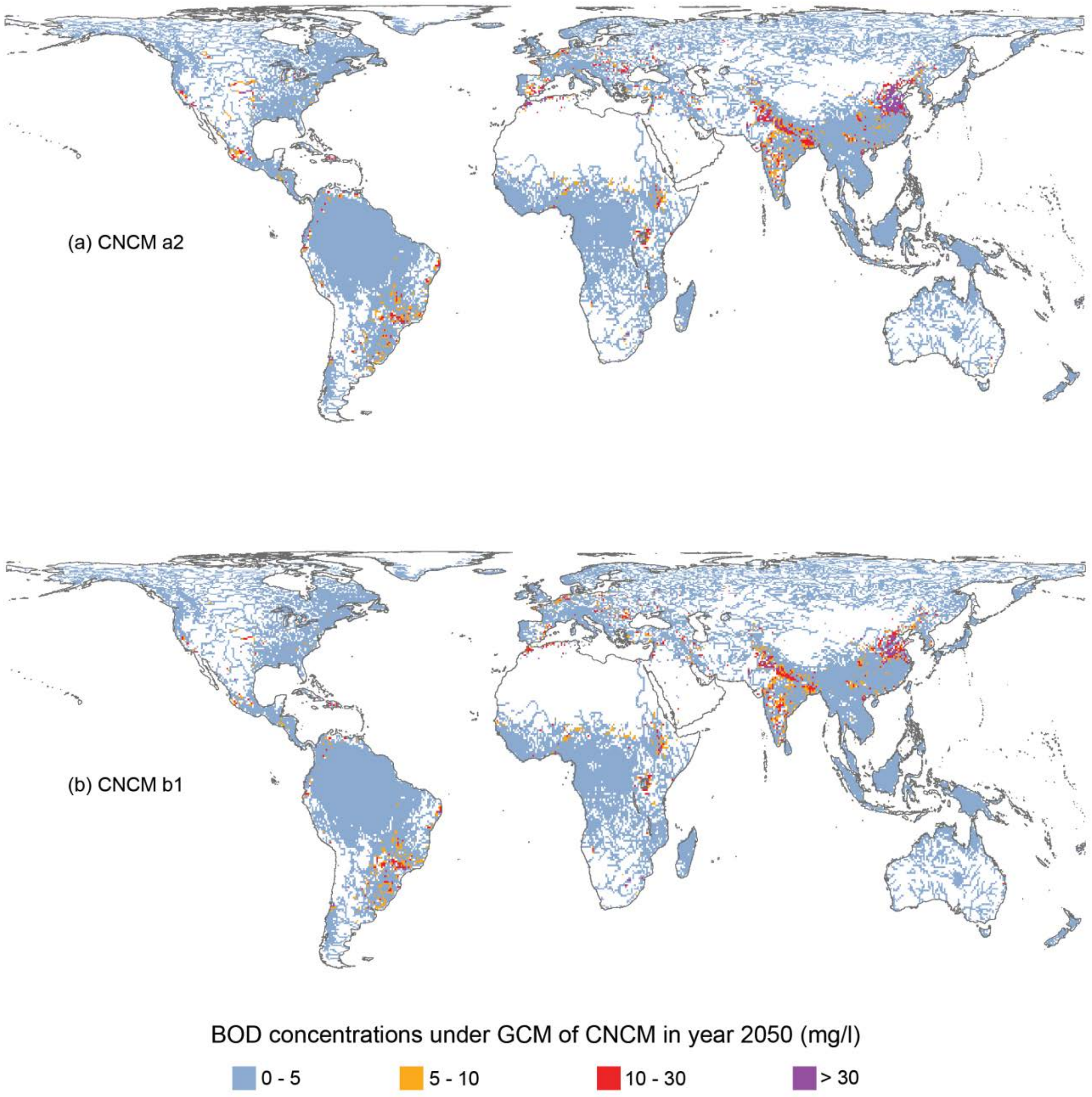

Figure S14. Simulated BOD concentrations using GCM of CNCM[31,32].

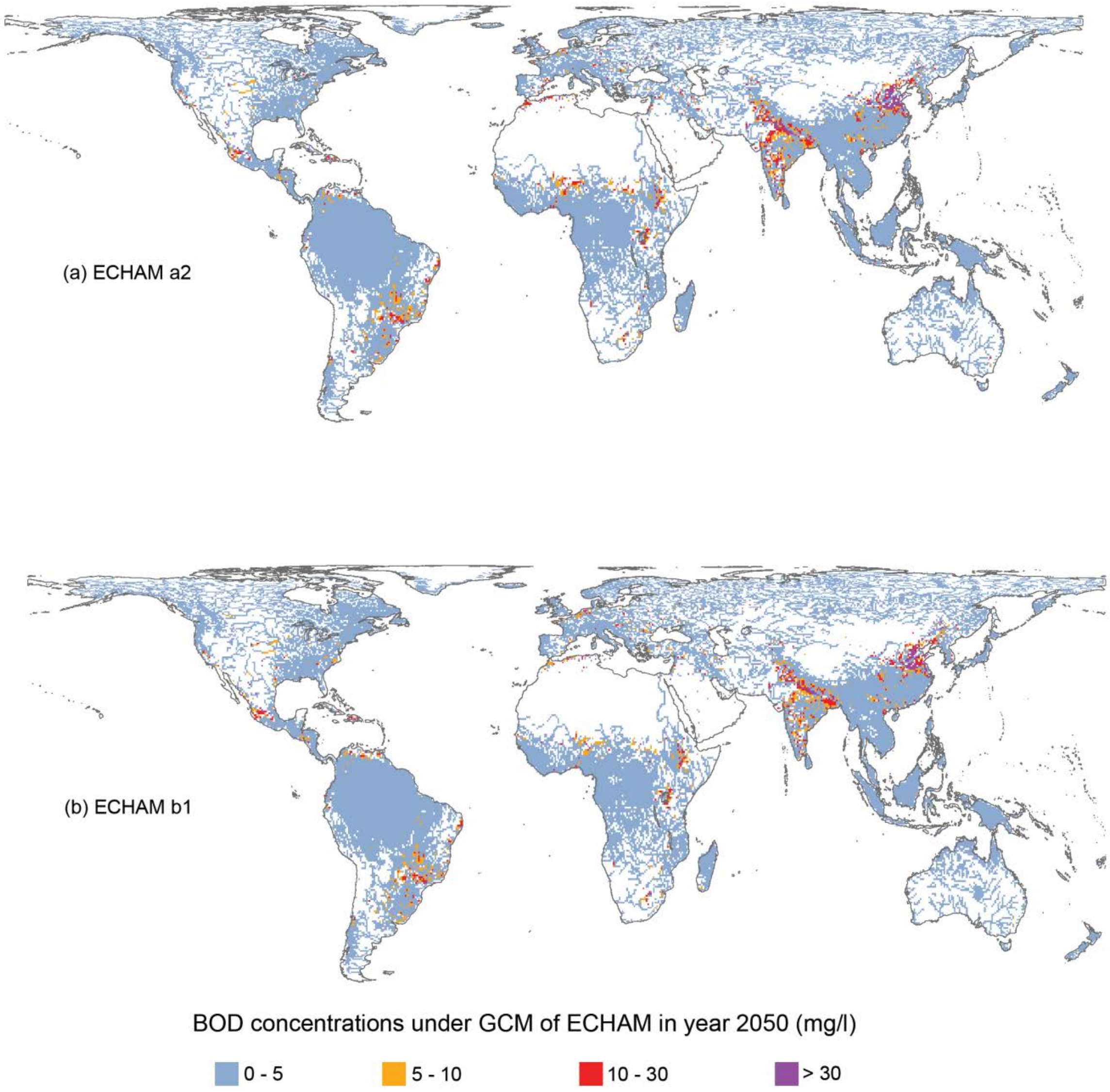

Figure S15. Simulated BOD concentrations in 2050 using GCM of ECHAM[31,32].

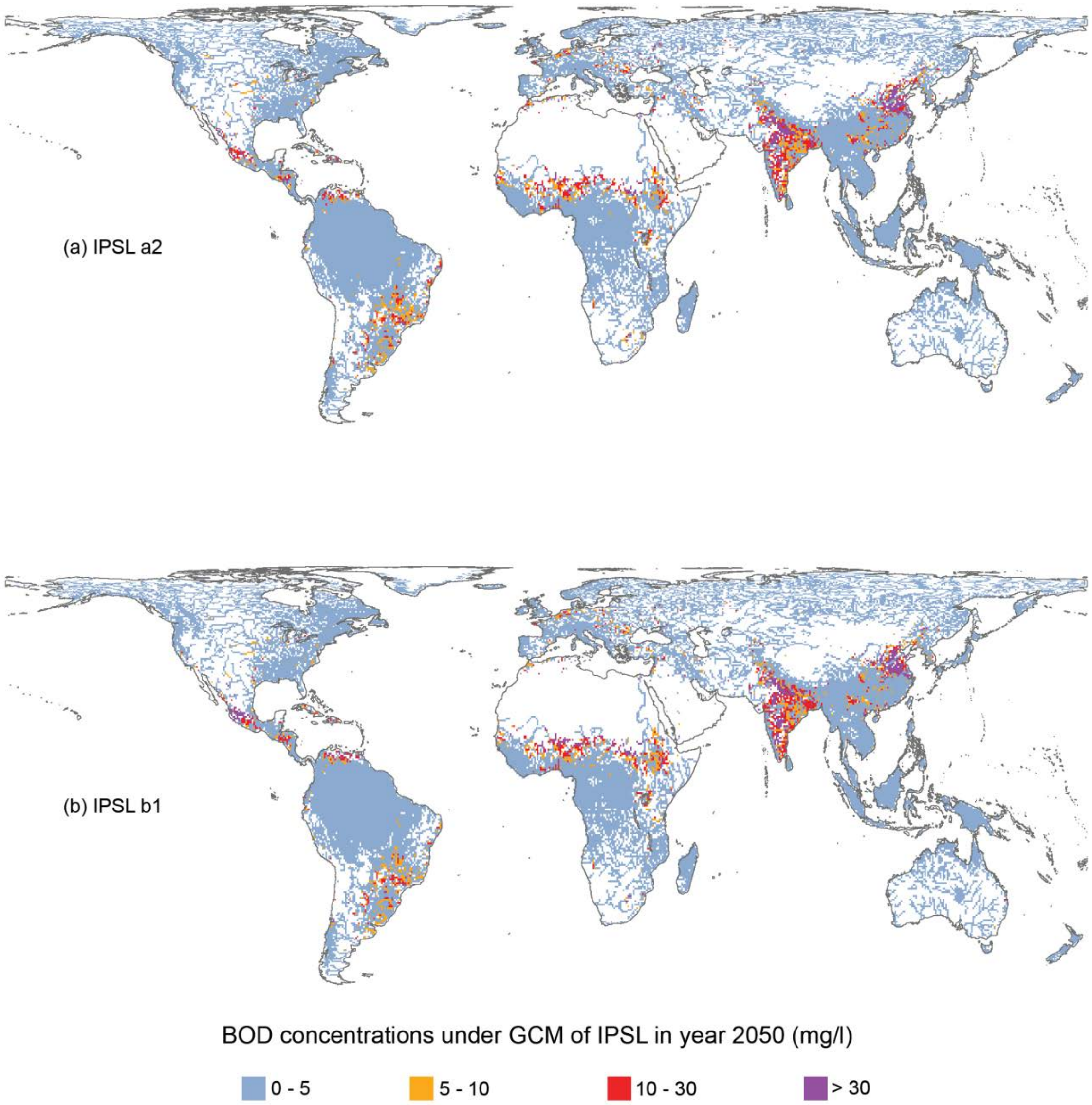

Figure S16. Simulated BOD concentrations in 2050 using GCM of IPSL[31,32].

Table S1. Overview of sources and methods for estimating model inputs.

Parameter	Symbol	Values	Sources
Upstream/downstream river segments	j/i	By grid cell	[1]
River discharge	Q_i	By grid cell	[2]
River length	X_j	By grid cell	[1]
River flow velocity	v_j	By grid cell	[4]
First-order rate coefficient for natural degradation	k	0.35 day^{-1}	[33]
Human BOD production	E_{hum}	By country	[5]
Urban population	P_i	By grid cell	[15,34]
Livestock animal population raised in intensive farming system	P_a	By grid cell	[35]
Livestock animal BOD production	E_a	By livestock type	[6]
Domestic wastewater treatment fraction	$f_{i,t}$	By city or country	[11,36]
Domestic wastewater treatment efficiency	$w_{i,t}$	By country	[7]
Intensive livestock farming treatment fraction	p_i	By region	[12]

Table S2. Urban BOD generation data for selected countries and regions[5].

Country/region	BOD (g/cap/day)
South Africa	40 ± 10
Zimbabwe	40 ± 10
Japan	55 ± 10
China	35 ± 10
Africa, Asia, Latin America, Caribbean	35 ± 10
Russia	50 ± 10
Europe	60 ± 10
United States	65 ± 10
Canada	60 ± 10
Australia and New Zealand	60 ± 10

Table S3. Livestock BOD generation data and animal mass equivalent coefficients[37].

	BOD (kg/1000 kg live animal mass/day)	Average animal mass equivalent coefficients	BOD (g/stock/day)
Buffalo & Cattle	1.6	1	400
Pig	3.1	0.3	233
Chicken	3.3	0.01	8.3

Table S4. Threshold density of intensive buffalo and cattle farming in different regions[37].

Region	Threshold density (TLUS/km^2)
Central and South America	29
East Asia	15
South Asia	61
Southeast Asia	13
Sub-Sahara Africa	8
West Asia and North Africa	10
others	25

Table S5. Urban wastewater treatment in India in the year 1999 (based on [8]).

	Metropolitan cities	Class I cities	Class II cities	Other cities
Population threshold	≥1,000,000	100,000 - 999,999	50,000 - 99,999	<50,000
% Total wastewater treatment	41	25	11	9
% Primary treatment	50	25	29	19
% Secondary treatment	50	75	71	38

Table S6. Ratios of wastewater treated to generated in urban and township areas in China in the year 2000[38].

	Urban	Town
National	28.6	7
East	55	7.7
West	25.8	4.5

Table S7. Urban wastewater treatment in Brazil in the year 2000[11,39,40].

	Class I	Class II	Class III	Class VI	Class V
Population threshold	> 300,000	100,000 – 300,000	45,000 – 100,000	20,000 – 45,000	< 20,000
% Total sewerage service	49	36	28	20	17
% sewerage treated	48	28	36	18	18

Table S8. Classification of BOD concentrations into six classes in China[22,23].

Class	I & II	III	IV	V	VI
BOD concentrations (mg/l)	≤3	3 - 4	4 - 6	6 -10	>10

Table S9. Comparison of calculated BOD concentrations to data from the GEMS global river water quality database, the European Waterbase database, the STORET Data Warehouse of the United States, Central Pollution Control Board of India, Ministry of Environmental Protection of the People's Republic of China and National Water Agency of Brazil presented as confusion matrix[41]. Values show the number of locations with observed and calculated concentrations in each of four categories. BOD concentrations above 5 mg/l indicate polluted water and above 10 mg/l require treatment before urban and agricultural reuse[42,43].

		Observed BOD concentration (mg/l)			
		0-5	5-10	10-30	>30
Calculated BOD concentration (mg/l)	0-5	654	33	5	0
	5-10	1	31	3	0
	10-30	0	0	28	0
	>30	0	0	0	8

Reference

1. Döll, P. & Lehner, B. Validation of a new global 30-min drainage direction map. *J. Hydrol.* **258,** 214–231 (2002).
2. Hydrology, C. for E. &. WATCH 21st century model output. (2011). Available at: http://www.waterandclimatechange.eu/about/watch-21st-century-model-output.
3. Fekete, B. M., Vörösmarty, C. J. & Grabs, W. High-resolution fields of global runoff combining observed river discharge and simulated water balances. *Global Biogeochem. Cycles* **16,** 15-1-15–10 (2002).
4. Schulze, K. & Hunger, M. Simulating river flow velocity on global scale. *Adv. Geosci.* **5,** 133–136 (2005).
5. U.S. EPA. *Quantification of Methane Emissions and Discussion of Nitrous Oxide and Ammonia from Septic Tanks, Latrines, and Stagnant Open Sewers in the World. U.S. Environmental Protection Agency* (EPA, 1999).
6. Practices, E. Manure Production and Characteristics American Society of Agricultural Engineers. *Am. Soc. Agric. Eng.* 682–685 (2003).
7. World Bank Group. Introduction to wastewater treatment processes. (2013). Available at: http://water.worldbank.org/shw-resource-guide/infrastructure/menu-technical-options/wastewater-treatment.
8. Raghupathi, U. P. *Status of Water Supply,Sanitaion and Solid Waste Management in Urban Areas*. (CPHEEO, 2005).
9. Ministry of Housing and Urban-Rural Development. *China Urban Waste Water Collection and Treatment Status Report 2006-2010.* (Ministry of Housing and Urban-Rural Development of China, 2010).
10. Balk, D. L. *et al.* Determining Global Population Distribution: Methods, Applications and Data. *Adv. Parasitol.* **62,** 119–156 (2006).
11. UNEP. *International Source Book On Environmentally Sound Technologies for Stormwater Management*. (UNEP, 2000).
12. Flörke, M. *et al.* Domestic and industrial water uses of the past 60 years as a mirror of socio-economic development: A global simulation study. *Glob. Environ. Chang.* **23,** 144–156 (2013).
13. Department of Economic and Social Affairs Statistics Division, U. N. *International Standard Industrial Classification of All Economic Activities, Rev.3.1*. (United Nations, 2008).
14. IPCC. *Climate change and water*. (IPCC, 2008).
15. United Nations. *World Urbanization Prospects The 2014 Revision*. (United Nations, 2014).
16. IPCC. *Emissions Scenarios*. (IPCC, 2000).
17. IFPRI. The International Model for Policy Analysis of Agricultural Commodities and Trade. (2015). Available at: http://impact-model.ifpri.org/.
18. GEMS/Water & UNEP. GEMStat. Available at: http://gemstat.org/default.aspx.
19. European Environment Agency. European Waterbase Database. (2014). Available at: http://www.eea.europa.eu/data-and-maps/data/waterbase-rivers-10.
20. U.S. EPA. The STORET Data Warehouse. (2013). Available at: http://ofmpub.epa.gov/storpubl/dw_pages.querycriteria.
21. National Water Agency, B. National Water Agency in Brazil. Available at: http://www2.ana.gov.br/Paginas/EN/default.aspx.
22. Ministry of Environmental Protection of the People's Republic of china. Weekly report of water quality on major rivers in China. (2000). Available at: http://datacenter.mep.gov.cn/.

23. China Ministry Environmental Protection. *Surface and drinking water standards*. (China Ministry Environmental Protection, 2002).
24. Mani, M. & Wheeler, D. In Search of Pollution Havens? Dirty Industry in the World Economy, 1960 to 1995. *J. Environ. Dev.* **7,** 215–247 (1998).
25. Torrecilla, N. J., Galve, J. P., Zaera, L. G., Retamar, J. F. & Álvarez, A. N. A. Nutrient sources and dynamics in a mediterranean fluvial regime (Ebro river, NE Spain) and their implications for water management. *J. Hydrol.* **304,** 166–182 (2005).
26. Fabian, D. B. P. *The Ebro River Basin*. *Water* **13,** (Springer Berlin Heidelberg, 2011).
27. Kengnal, P., Megeri, M. N., Giriyappanavar, B. S. & Patil, R. R. Multivariate Analysis for the Water Quality Assessment in Rural and Urban Vicinity of Krishna River (India). *Asian J. Water, Environ. Pollut.* **12,** 73–80 (2015).
28. Aba, K. D. & Dasharath, G. D. ROLE OF MIGRATORY LABOUR (WOMEN AND MEN) IN DEVELOPMENT OF SUGAR INDUSTRY IN UPPER KRISHNA VALLEY. *Int. J. Entrep. Bus. Environ. Perspect.* **2,** 272 (2013).
29. Cohen, J. E. Human Population: The Next Half Century. *Science (80-.).* **302,** 1172–1175 (2003).
30. Department of Economic and Social Affairs Statistics Division, U. N. *World population prospects: The 2012 revision*. *United Nations* (United Nations, 2013).
31. Esri. ArcMap 10.1. http://www.esri.com/
32. MathWorks. MatLab R2012b. http://nl.mathworks.com/index.html?s_tid=gn_logo
33. Thomann, R. V. & Mueller, J. A. *Principles of surface water quality modeling and control*. (Waveland press, 1987).
34. Balk, D., Yetman, G. & Sherbinin, A. De. Construction of gridded population and poverty data sets from different data sources. in *European Forum for Geostatistics Conference* 5–7 (2010).
35. FAO. The Gridded Livestock of the World (GLW). Available at: http://www.fao.org/ag/againfo/resources/en/glw/GLW_dens.html.
36. Van Drecht, G., Bouwman, a. F., Harrison, J. & Knoop, J. M. Global nitrogen and phosphate in urban wastewater for the period 1970 to 2050. *Global Biogeochem. Cycles* **23,** (2009).
37. FAO. *Global livestock production systems*. (2011).
38. Ministry of Housing and Urban-Rural Development. *China Urban Waste Water Collection and Treatment Status Report 2000-2004*. (Ministry of Housing and Urban-Rural Development of China, 2005).
39. Marcon, G. & Philippi, A. Analysis of basic sanitation in Brazil and its impact on water resources and health. *Rega* **7,** 61–70 (2010).
40. IBGE. *Pesquisa Nacional de Saneamento Básico 2000*. (Instituto Brasileiro de Geografia e Estatística, 2002).
41. Fawcett, T. An introduction to ROC analysis. *Pattern Recognit. Lett.* **27,** 861–874 (2006).
42. Abbasi, T. & Abbasi, S. A. *water quality indices*. (Elsevier, 2012).
43. U.S. EPA. *2012 Guidelines for Water Reuse*. (U.S. EPA, 2012).
